# Response of *Ustilago maydis* against the Stress Caused by Three Polycationic Chitin Derivatives

**DOI:** 10.3390/molecules22121745

**Published:** 2017-12-07

**Authors:** Dario Rafael Olicón-Hernández, Cristina Uribe-Alvarez, Salvador Uribe-Carvajal, Juan Pablo Pardo, Guadalupe Guerra-Sánchez

**Affiliations:** 1 Instituto Politécnico Nacional, Escuela Nacional de Ciencias Biológicas, Departamento de Microbiología, Prolongación de Carpio y Plan de Ayala S/N, Col. Sto. Tomas, Del, Miguel Hidalgo, CP 11340 Ciudad de México, Mexico; magnadroh@hotmail.com; 2 Universidad Nacional Autónoma de México, Instituto de Fisiología Celular, Circuito exterior S/N, Ciudad Universitaria, CP 04510 Ciudad de México, Mexico; curibe@email.ifc.unam.mx (C.U.-A.); suribe@ifc.unam.mx (S.U.-C.); 3 Universidad Nacional Autónoma de México, Facultad de Medicina, Departamento de Bioquímica, Circuito exterior S/N, Ciudad Universitaria, CP 04510 Ciudad de México, Mexico; pardov@bq.unam.mx

**Keywords:** chitosan, oligochitosan, glycol-chitosan, *Ustilago maydis*, stress response

## Abstract

Chitosan is a stressing molecule that affects the cells walls and plasma membrane of fungi. For chitosan derivatives, the action mode is not clear. In this work, we used the yeast *Ustilago maydis* to study the effects of these molecules on the plasma membrane, focusing on physiologic and stress responses to chitosan (CH), oligochitosan (OCH), and glycol-chitosan (GCH). Yeasts were cultured with each of these molecules at 1 mg·mL^−1^ in minimal medium. To compare plasma membrane damage, cells were cultivated in isosmolar medium. Membrane potential (Δψ) as well as oxidative stress were measured. Changes in the total plasma membrane phospholipid and protein profiles were analyzed using standard methods, and fluorescence-stained mitochondria were observed. High osmolarity did not protect against CH inhibition and neither affected membrane potential. The OCH did produce higher oxidative stress. The effects of these molecules were evidenced by modifications in the plasma membrane protein profile. Also, mitochondrial damage was evident for CH and OCH, while GCH resulted in thicker cells with fewer mitochondria and higher glycogen accumulation.

## 1. Introduction

Ecological niches for fungi are numerous and varied. In nature, fungi can be found as saprophytic agents, as pathogenic or phytopathogenic parasites, as communities (mycorrhiza), or as symbionts [1]. Thus, fungi are exposed to different environmental stress conditions such as variations in pH, temperature, osmolarity, toxins, and natural or synthetic compounds that could damage their structure or disrupt their metabolism and yet, they survive [2]. Stress in fungi may be an external biotic or abiotic condition that interferes with optimal growth parameters and generates physiological responses [3]. These defense responses may involve overexpression of genes related to carbohydrate metabolism [4], the production of structural proteins [5], modifications in cell wall or membrane composition [6,7], changes in cell integrity [4], and the production of reactive oxygen species (ROS) [8]. One of the advantages of studying stress responses in fungi is that they are excellent models of eukaryotic responses against external or internal factors which could also be observed in plants and animals; indeed, very conserved defense mechanisms exist [9].

Chitosan (CH) elicits a strong stress response in fungi. It is a polycationic semi-natural carbohydrate with an average molecular weight of 50 kDa, constituted by the aminated sugars glucosamine (<90%) and *N*-acetyl glucosamine (>10%) [10]. The main source of chitosan is their extraction from chitin from insects and crustaceans; however, it also is an important constituent of green algae, yeasts, protozoa, as well as the cell walls of some fungi and used in the fabrication of environmentally friendly antimicrobial agents [11]. In this context, it was reported that CH and its derivatives are able to inhibit bacteria, fungi, and viruses that can generate diseases from the clinical and environmental point of view, mainly by boosting the immune systems of humans, animals, and plants, interfering with the normal metabolism of the pathogens, destroying cellular structures [12,13,14]. In acidic pH, the CH amino groups are protonated and thus, CH is polycationic. It is used to produce different antimicrobial agents [15], such as oligochitosan (OCH) and glycol-chitosan (GCH), also polycationic in nature, which are applied in different industries and research activities. The OCH (MW around 5 kDa) is smaller than chitosan [16], while GCH is produced by etherification of chitosan and ethylene glycol and is used in bio-gel fabrication [12]. In contrast to CH, which is soluble only at an acid pH, both OCH and GCH are soluble at pH 7.0. The polycationic nature of these compounds allows strong interactions with different fungal structures such as the cell wall, membrane lipids, proteins, and nucleic acids, probably triggering a stress response [17]. In eukaryotic cells, chitosan and its derivatives promote morphological mechanical alterations [15], increased ROS concentration [18,19], mitochondrial dysfunction [20], decreased metabolic processes [21], decreased septation, increased spore mitoses [22], and overexpression of genes related to oxidative stress [23]. Fungi react to these polycations through proteins involved in plasma membranes, respiration, ATP production, and mitochondrial organization. In *Neurospora crassa,* the generation of reactive oxygen species, cellular energy, and cellular membrane homeostasis was affected by chitosan [21].

*Ustilago maydis* is a basidiomycete and an ustilaginal fungus used as a model species in several biochemical and physiological studies. Due to its accessible genome, easy handling, ability to grow in defined media by budding and formation of compact colonies on plates, *U. maydis* is considered an ideal fungal model for cell and molecular biology studies [24]. We have previously described the effects of CH, OCH, and GCH on the development and morphology of *Ustilago maydis* [15]. Here, we characterize the response of *U. maydis* to each of these polycationic compounds and conclude that each chitosan derivative triggers a specific stress-like response in *U. maydis*.

## 2. Results

### 2.1. Growth of U. maydis at Different Osmolarities: Effects of CH, OCH, and GCH

**Figure 1:**
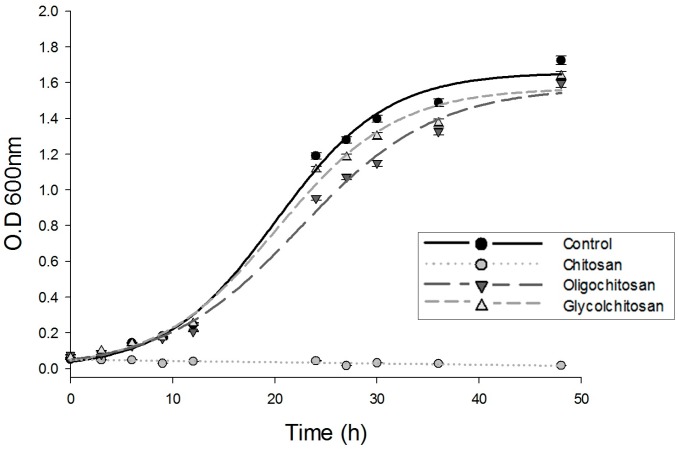
Growth of *Ustilago maydis* in minimal medium and in the presence of chitosan, oligochitosan or glycol-chitosan. Each agent was added to a final concentration of 1 mg·mL^−1^. Growth was measured as an increase in optical density at 600 nm.

In hypo-osmotic medium, CH inhibits the growth of *U. maydis*, while OCH and GCH have no effects [15]. Here, when culturing the cells in an isosmotic medium, the same inhibition pattern was observed (Figure 1), i.e., OCH and GCH did not exhibit any effect, while CH fully inhibited *U. maydis* growth.

### 2.2. Cell Membrane Permeability Changes in U. maydis upon Addition of CH, OCH or GCH

**Figure 2:**
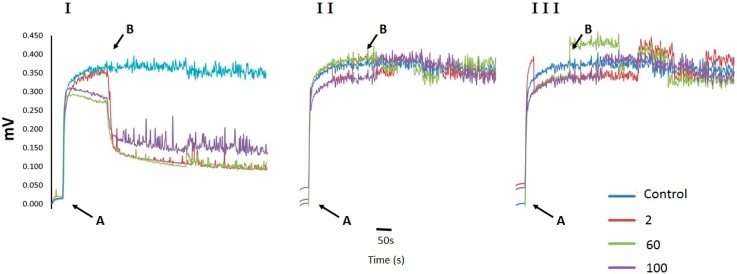
Changes in Δψ in response to different concentrations of CH, OCH or CGH. All concentrations are expressed in µg·mL^−1^. The numbers correspond to each antifungal tested, (**I**) CH; (**II**) OCH; (**III**) GCH. Additions were: A = cells; B = Antifungal. *U. maydis*, 25 mg wet weight.

Since osmotic support to *U. maydis* did not prevent the chitosan effect, it was decided to determine whether the plasmatic membrane was intact. For this, the transmembrane electrical potential (Δψ) in the cell was estimated using a cyanine derivative [25]; 2 µg·mL^−1^ of chitosan decreased Δψ by more than 50%. Higher concentrations further decreased Δψ (Figure 2I). The presence of different polycation compounds (OCH) and (GCH) did not produce changes in this physiological parameter (Figure 2II,III).

### 2.3. Determination of ROS Released by U. maydis as a Response to CH, OCH or GCH

**Figure 3:**
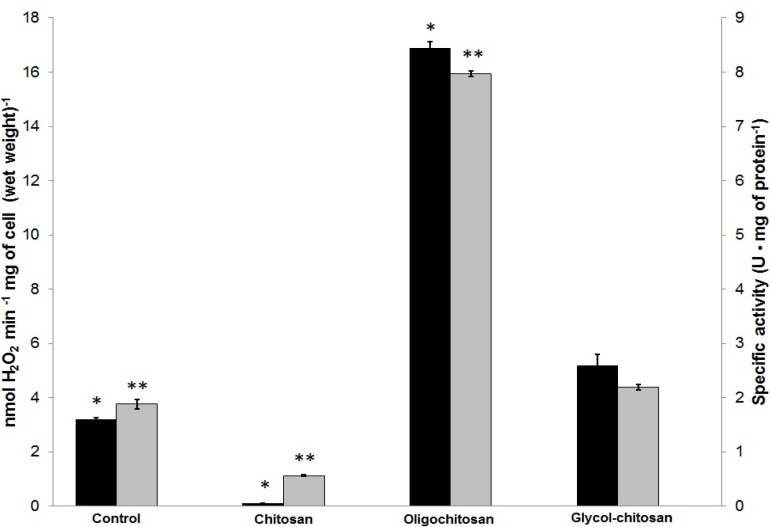
Quantification of ROS production by *Ustilago maydis* grown in the presence of CH, OCH or GCH. H_2_O_2_ production as measured with Amplex red^®^ (■) and catalase activity (■). Cells were incubated in minimal medium for 24 h at 128 rpm and the indicated antifungal agent was added at 1 mg·mL^−1^. Significance was evaluated by one-way ANOVA analysis and Tukey test (*p* < 0.05). The experiments were performed in triplicate (*n* = 3). * and ** indicate significant difference in H_2_O_2_ production and catalase activity, respectively, compared to control cells.

The production/presence of ROS was measured directly (Amplex Red^®^ method) and indirectly (catalase activity) after treatment with the different polycation compounds. During growth, the control cells produced 3.13 nmol H_2_O_2_ min^−1^ mg wet weight^−1^, and the catalase-specific activity was 1.87 U mg protein^−1^, indicating that some hydrogen peroxide is formed under the physiological conditions and normal growth. Cells treated with chitosan produced considerably less ROS (0.11 nmol H_2_O_2_ min^−1^ mg of cell wet weight^−1^) and showed a catalase activity of 0.56 U mg of protein^−1^. This is associated with immediate and total CH-mediated cell destruction [15]. The OCH treatment produced a statistically significant increase in ROS (16.6 nmol H_2_O_2_ min^−1^ mg of cell wet weight^−1^ and catalase activity of 7.97 U mg of protein^−1^), which means that it produced a greater amount of ROS compared to the other treatments. Cells grown in GCH did not show any differences in terms of ROS production (5.75 nmol H_2_O_2_ min^−1^ mg of cell wet weight^−1^ and 2.19 U mg of protein^−1^) compared to the control cells; this was corroborated by the results of Tukey´s test (Figure 3).

### 2.4. CH-, OCH-, or GCH-Mediated Damage of the Mitochondrial Structure in U. maydis

**Figure 4:**
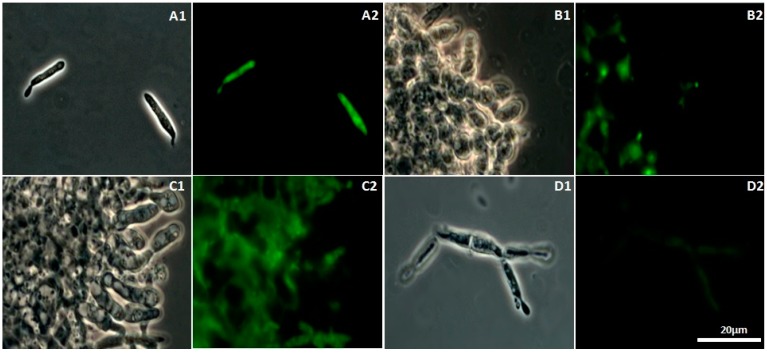
Phase contrast (1) and fluorescence (2) microscopy of *U. maydis* stained with MitoTracker^®^ Green FM. An Eclipse E800 fluorescent microscope (Nikon Instruments Inc., Melville, NY, USA) with a fluorescein filter was used. The cells were growth in MM with the different treatment in a concentration of 1 mg mL^−1^. The intensity of the signal is according with the presence of the mitochondrial proteins. **A** = Control; **B** = Chitosan; **C** = Oligochitosan; **D** = Glycol-chitosan. Time of incubation of 24 h.

Damages in the mitochondrial structure were observed by the use of Mitotracker green^®^ (Invitrogen/Thermo Fisher Scientific, Waltham, MA, USA) via fluorescence microscopy. In all cases, background fluorescence was observed, but specific regions were stained with greater intensity when mitochondria were present, especially in the control cells (Figure 4A2). The mitochondrial staining of cells with CH and OCH (Figure 4B2,C2) showed irregular fluorescence distribution, suggesting high damage. Cells incubated in the presence of GCH exhibited a lower fluorescence intensity than the control cells (Figure 4D2), suggesting mitochondria loss.

### 2.5. Chitin Derivatives Modify Total Phospholipid Contents

As shown in Table 1, the phospholipid contents of the membrane fraction of yeast grown in the presence of chitin derivates were statistically decreased. The greatest decrease (44.72%) was induced by oligochitosan, followed by glycol-chitosan with an approximated decrease by 17.78% compared to the control. There was no data for chitosan, due to its inhibitory effect on yeast growth.

### 2.6. SDS-PAGE Analysis of U. maydis Membrane Proteins in the Absence and Presence of CH, OCH or GCH

**Figure 5:**
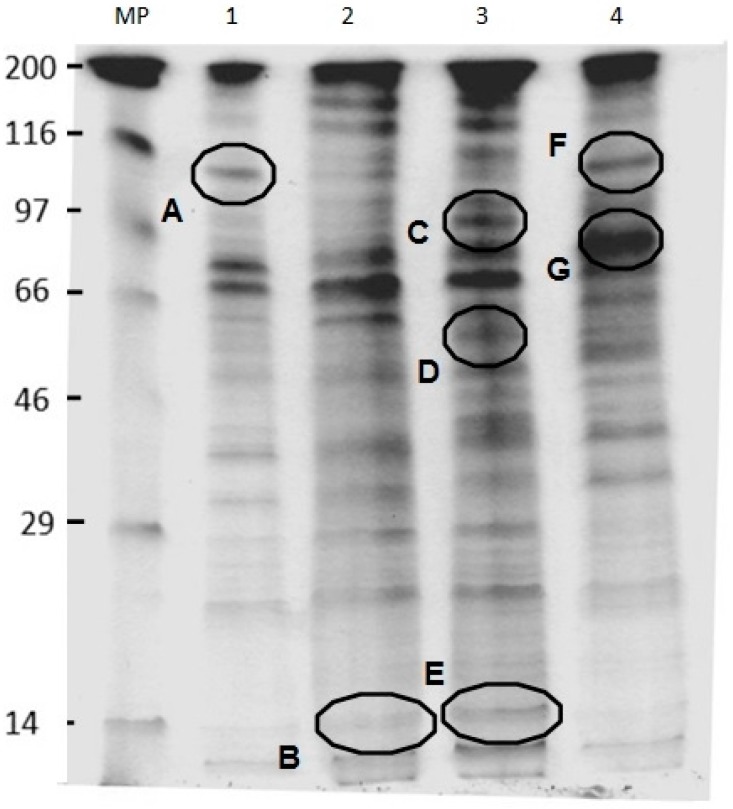
SDS-PAGE of the membrane fraction of *U. maydis* after the different antifungal treatments. Oligochitosan and glycol–chitosan were added at 1 mg·mL^−1^. Chitosan was added at 10 µg·mL^−1^ due to the effect on cell growth. MW= Molecular weight marker (KDa); 1 = Control cell without antifungals; 2 = Cells treated with chitosan; 3 = Cells treated with oligochitosan; 4 = Cells treated with glycol–chitosan. Squares indicate differences in the bands pattern.

The SDS-PAGE analysis (Figure 5) was performed to observe the modification in the protein profile caused by the addition of chitin derivatives. In CH-treated cells, the decrease in the intensity of a band near 100 kDa, suggested as the electrophoretic mark of the plasma membrane H ± ATPase (band A) [26], was observed. The samples treated with OCH exhibited the largest changes, presenting a more intense protein band at approximately 90 kDa (band C), 57 kDa (Band D), and 14 kDa (band E). The A band was not neatly distinguished as observed in the control cells. Cells grown in glycol–chitosan showed a well-delimited band A, with greater banding intensity in a protein of near 83 kDa (Band G).

### 2.7. Effects of CH, OCH or GCH on the Accumulation of Glycogen by U. maydis

**Figure 6:**
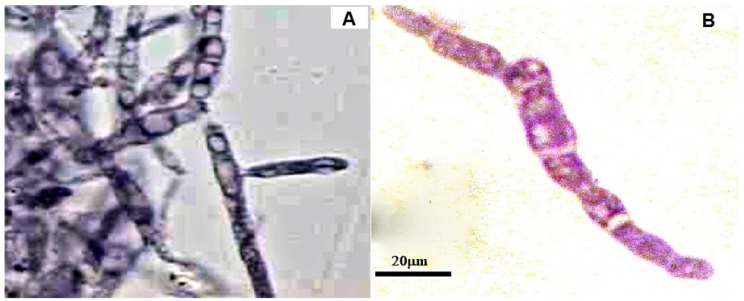
PAS staining of *U. maydis* in the presence of GCH. A 1 mg·mL^−1^ of each antifungal compound was added. The positive schiff reaction was demonstrated by the presence of the purple compound derivate of the interaction between the basic fuchsin with aldehydes sugars. (**A**) = Control; (**B**) = Glycol-chitosan (100×).

In response to stress, bacteria and yeasts accumulate glycogen. The cells cultured in the presence of GCH accumulated glycogen as compared to the control (Figure 6A). No glycogen was detected by PAS staining of cells with CH and OCH (data not shown). Interestingly, cells with GCH showed an increased size compared to the control (Figure 6B). This abnormal glycogen accumulation suggests that metabolic changes may be activated to use the GCH as fuel storage, which induces cell thickening.

## 3. Discussion

Adverse external conditions trigger the stress defense response that comprises diverse metabolic modifications needed for survival [27]. In *U. maydis*, different chitin derivatives prompted different responses. As our results show, high osmolarity did not modify the effects of the different derivatives tested, indicating that the findings observed for the fungus do not solely result from osmotic stress [15]. This observation stands in contrast to the evidence reported by Zakrzewska et al. [28], who observed that 1M sorbitol protected *Saccharomyces cerevisiae* cells against chitosan. The authors state that the high-osmolarity glycerol pathway is crucial to establish fungal sensibility to chitosan. In this regard, *U. maydis* does exhibit important differences in the activation and control of this pathway, which would explain its sensitivity to chitosan, even in an isosmolar environment [28,29]. As reported before, chitosan promotes has a complete inhibitory effect on *Ustilago maydis* as a consequence of the interaction between specific sites in the plasma membrane and protonated free amino groups, constituting the main change of the polymer [15]. The modifications in cell permeability explain the depletion of the membrane potential and are consistent with observations in *Rhizopus stolonifer* at a chitosan concentration of 2000 µg·mL^−1^ [15,30]. However, in a non-susceptible model, such as *Candida albicans*, the effect of chitosan on the plasma membrane is completely the opposite: Peña et al. [31] described hyperpolarization of the cell membrane of *C. albicans* due to an alignment of internal charges with a subsequent increase in potential membrane when the yeast was in the presence of low chitosan concentrations [31]. Thus, it is evident that the effects of chitosan vary depending on the model [32]. In this study, OCH and GCH did not affect the plasma membrane potential.

There is evidence of an enhancement of oxygen consumption in *R stolonifer*, *C. albicans*, and *U. maydis* by chitosan and oligochitosan [15,31,33]. This modification of the rate of oxygen consumption could be due to an increased use of ATP, involved in energy-depended defense against these polymers, or to the generation of reactive oxygen species [31]. Oxidative stress in the presence of OCH has previously been described in macrophages, plant cells, and fungi [16,18,34]. In the last case, it was proposed that chitin derivatives inhibited proteins involved in the generation of reducing power for the neutralization of intracellular ROS, such as glutathione S-transferase-4 [34]. Our results do not indicate whether the formation of ROS is part of the oligochitosan antifungal effect or simply a response to the stress generated by these molecules. On the other hand, the addition of CH and OCH seems to disorganize the mitochondrial structure without affecting its function. In contrast, in *U. maydis*, GCH does affect mitochondrial function. Previously, we described the reduction in the oxygen consumption rate in *U. maydis* in the presence of GCH [15]. In the present work, we corroborated the affectation of the mitochondria by GCH; this result is consistent with the decrease in respiration in *U. maydis* by the addition of glycol-chitosan, which has been described previously [15]. We believe this is the first report on the mitochondrial effects of GCH.

In the presence of chitin derivatives, the responses of *U. maydis*, modifying its lipid, protein, and carbohydrate composition, are part of a global survival mechanism. The OCH modified the total phospholipids and, to a lesser extent, so did GCH. Additionally, the protein profile was modified by the presence of each of the compounds used. The stressors may interact with the plasma membrane and produce different signals that up-regulate different membrane proteins. The modification of the protein concentration and protein functionality during CH stress has been described for *R. stolonifer*, where the amount of membrane proteins decreases to about 50%, as well as the activity of H + ATPase [35]. Under several stress conditions, fungi are able to modify the composition of their cell membrane, accumulate glycogen as a secondary energy source, and express, overexpress, and/or repress several genes for kinases, enzymes, transcriptional factors, detoxification systems, and mediators of apoptosis [36,37,38,39,40,41]. In fungi, chitin derivatives increased proteins for ergosterol synthesis, actin cytoskeleton organization, protein *N*-glycosylation, endocytosis, cell wall formation, and carbohydrate metabolism [28]. Previously, it has been demonstrated that chitosan produces a fungal stress response, involving a large number of genes mediated by the action of common stress transcription factors in yeast, such as Msn2p and Msn4p [42]. It is therefore possible that in *U. maydis*, the same factors are involved in the stress response to CH, OCH, and GCH.

Our results demonstrated that chitosan and its derivatives produce stress responses in *U. maydis*; these responses are significantly different depending on the characteristics of the molecule. Further experiments are necessary to establish the mode of action of these polycationic compounds and the stress response they elicit in fungi. 

## 4. Materials and Methods

### 4.1. Reagents and Solutions

Low molecular weight chitosan (deacetylation degree (DD) ≥ 85%, MW 50–190 kDa), oligochitosan (chitosan oligosaccharide lactate, DD > 90%, average MW 5 kDa), and glycol chitosan (DD ≥ 60%, average MW 250 kDa) were purchased from Sigma-Aldrich (St. Louis, MO, USA). The stock solutions of CH, OCH, and GCH were prepared according to our previously published protocol [15]. All chemical compounds and solvents were analytical grade.

### 4.2. Growth in Isosmolar Medium

*Ustilago maydis* ATCC 201384 FB2 was grown in minimal medium (1% glucose, 0.3% potassium nitrate, and salt solution, pH 5.6), individually or in the presence of 1 mg CH, OCH, or GCH per mL. To establish an isosmolar environment, 400 mM sorbitol was added. This isosmolar medium was prepared considering the intracellular potassium concentration and its counter anions of the model yeast *Saccharomyces cerevisiae* [43]. Cells were cultured at 28 °C under agitation at 130 rpm for 48 h. Growth was measured by the changes in optical density at 600 nm.

### 4.3. Transmembrane Potential

Yeasts of *U. maydis* were cultured in YPD (1% yeast extract, 0.15% ammonium nitrate, 0.25% bacto peptone, 1% glucose, pH 6.8) for 24 h under the condition previously described [15]. Biomass was centrifuged at 3000× *g* for 10 min and diluted to 50% (*w/v*) with distilled water; 10-µL samples were used. The membrane potential was estimated by following the changes in fluorescence of a 0.25 mM cyanine solution at 540–590 nm, according to the protocol previously described by Peña et al. [25]. Increasing concentrations of each chitosan derivative (from 2 to 100 µg·µL^−1^) were added after 50 s and the effects on the transmembrane potential were monitored.

### 4.4. H_2_O_2_ Production Measured by the Amplex Red^®^ Method

Cells were cultured (24 h/130 rpm/28 °C) in minimal media and with or without 1 mg polycation mL^−1^. The cells were collected by centrifugation and a 50% (*w/v*) cell suspension was prepared with sterilized water. Subsequently, 20-µL aliquots were placed into an ELISA 100-well plate with 50 µL of reaction mixture (0.1 units mL^−1^ horseradish peroxidase, 100 units mL^−1^ superoxide dismutase, 10 µM Amplex red^®^ (Thermo Fisher Scientific, Waltham, MA, USA), 0.6 M mannitol, and 5 mM MES) and the volume was adjusted to 100 µL. The mixture was incubated at room temperature for 5 min. The formation of the fluorescence derivative (resorufin) as a consequence of the release of H_2_O_2_ was measured in a PolarStar OMEGA detector (571–585 nm), using the OMEGA control software (Ortenberg, Germany); the results were interpolated against a calibration curve [44]. The data are reported as nmol of H_2_O_2_ per min per mg wet weight of cells.

### 4.5. Catalase Activity

We also used *U. maydis* to measure catalase in cells grown in the absence or presence of each chitosan derivative. Cells were collected by centrifugation and mechanically disrupted in 0.1 M phosphate buffer (pH 7), using 0.5 mm glass beads. Subsequently, 100 µL of the supernatant were taken and added to 1.5 mL phosphate buffer. After this, 1 mL of 5 mM hydrogen peroxide was added and catalase activity was measured by the decrease in absorbance at 240 nm after 10 min. Protein was quantified by the Lowry method [45]. The specific activity of catalase is reported as units per mg protein. One unit is defined as the amount of enzyme necessary to reduce the absorbance at a rate of 0.1 units per mL per minute [46].

### 4.6. Mitochondrial Staining

*Ustilago maydis* was grown in minimal medium supplemented with 1 mg·mL^−1^ of each compound for 24 h, under the conditions described before. Cell suspension aliquots of 1 mL were incubated in the presence of 25 nM Mitotracker green^®^ (Invitrogen/Thermo Fisher Scientific, Waltham, MA, USA) for 30 min at 37 °C. Untreated cells were used as control. Fluorescent mitochondria were observed in phase contrast microscopy with a fluorescein filter (494–520 nm) [47].

### 4.7. Total Phospholipid Quantification

The membrane fraction of *U. maydis* grown in the presence of each compound at a concentration of 1 mg·mL^−1^ for 24 hours at 28 °C was obtained by fractional centrifugation, as described in our previous publication [15]. For the quantification of total phospholipids, a commercial kit was used (Spinreact, Spain). Phospholipid concentration was determined by comparing the absorbance against a standard containing 300 mg·dL^−1^ of total phospholipids. The results were expressed in mM phospholipids g of cells^−1^ (wet weight).

### 4.8. SDS-PAGE of the Membrane Fraction

Cells were incubated for 24 h on a shaker in the presence of each compound at 1 mg·mL^−1^, with the exception of CH, which was added at 10 µg·mL^−1^. The membrane fraction was obtained and the Lowry method was used to determine the protein concentration. Samples with ≈30 µg of protein were mixed with 4× loading buffer (0.5 M Tris pH 6.8; 10% SDS; 15% β-mercaptoethanol, 25% glycerol, and 0.1 mg·mL^−1^ bromophenol blue) and analyzed on 10% SDS-PAGE; staining and de-staining were performed with Coomassie Blue and acetic acid–methanol–water (1:2:10) solution, respectively.

### 4.9. Glycogen Accumulation and PAS Staining 

*Ustilago maydis* was fixed with 0.6% periodic acid solution for 10 min at room temperature. The sample was washed with distilled water and mixed with Schiff's reagent (leucobasic fuchsin) for 30 min. Subsequently, the samples were washed three times with 10% sodium metabisulfite-1 N HCl for 1 min. Samples were rinsed with an ethanol-xylene mixture, mounted on a slide with synthetic resin, and observed in an optical microscope, denoting a purple color in the case of glycogen presence.

## Figures and Tables

**Table 1:** Total phospholipid concentration (mM phospholipids g of cells^−1^) in the membrane fraction of *U. maydis* growth under antifungal treatments.

Antifungal Tested (1mg·mL^−1^)	Total Phospholipid Concentration (mM Phospholipids g of Cells^−1^ Wet Weight)
Control	4.54 ± 0.035 ^a^
Chitosan	ND
Oligochitosan	2.51 ± 0.12 ^b^
Glycol-chitosan	3.76 ± 0.043 ^c^

ND = Not determined. Different letters (a, b and c) indicate significant difference in one-way ANOVA evaluated by Tukey test (*p* < 0.05). The experiments were performed in triplicate (*n* = 3).
